# The Reliability of Pharyngeal High Resolution Manometry with Impedance for Derivation of Measures of Swallowing Function in Healthy Volunteers

**DOI:** 10.1155/2016/2718482

**Published:** 2016-04-14

**Authors:** Taher I. Omari, Johanna Savilampi, Karmen Kokkinn, Mistyka Schar, Kristin Lamvik, Sebastian Doeltgen, Charles Cock

**Affiliations:** ^1^Department of Gastroenterology, Flinders Medical Centre and School of Medicine, Flinders University, Adelaide, SA 5042, Australia; ^2^Human Physiology, Medical Science and Technology, School of Medicine, Flinders University, Adelaide, SA 5042, Australia; ^3^Department of Anaesthesiology and Intensive Care, Örebro University Hospital and School of Medical Sciences, Örebro University, Örebro, Sweden; ^4^Swallowing Rehabilitation Research Laboratory, The University of Canterbury Rose Centre for Stroke Recovery and Research, Christchurch, New Zealand; ^5^Discipline of Speech Pathology & Audiology, Flinders University, Adelaide, SA 5042, Australia

## Abstract

*Purpose*. We evaluated the intra- and interrater agreement and test-retest reliability of analyst derivation of swallow function variables based on repeated high resolution manometry with impedance measurements.* Methods*. Five subjects swallowed 10 × 10 mL saline on two occasions one week apart producing a database of 100 swallows. Swallows were repeat-analysed by six observers using software. Swallow variables were indicative of contractility, intrabolus pressure, and flow timing.* Results*. The average intraclass correlation coefficients (ICC) for intra- and interrater comparisons of all variable means showed* substantial* to* excellent* agreement (intrarater ICC 0.85–1.00; mean interrater ICC 0.77–1.00). Test-retest results were less reliable. ICC for test-retest comparisons ranged from* slight* to* excellent* depending on the class of variable. Contractility variables differed most in terms of test-retest reliability. Amongst contractility variables, UES basal pressure showed* excellent* test-retest agreement (mean ICC 0.94), measures of UES postrelaxation contractile pressure showed* moderate* to* substantial* test-retest agreement (mean Interrater ICC 0.47–0.67), and test-retest agreement of pharyngeal contractile pressure ranged from* slight* to* substantial *(mean Interrater ICC 0.15–0.61).* Conclusions*. Test-retest reliability of HRIM measures depends on the class of variable. Measures of bolus distension pressure and flow timing appear to be more test-retest reliable than measures of contractility.

## 1. Introduction

In dysphagia of broad aetiology, the accurate diagnosis of pathophysiology underlying swallowing dysfunction is critical for providing appropriately targeted treatments. Given the mechanical complexity of swallowing, this has been a major challenge. High resolution solid state manometry (HRM) or HRM with impedance (HRIM) are catheter-based diagnostic modalities that are gaining increasing interest as a potential adjunct method for the assessment of pharyngeal function in patients with dysphagia symptoms. The equipment to perform HRM/HRIM investigations is now widely available. It is also mobile and therefore amenable to use at the bedside or in a community clinic setting. Used as an adjunct to videofluoroscopy, HRM/HRIM may improve diagnosis by introducing biomechanically based evaluations of swallowing into the diagnostic paradigm [1].

The pressure and flow values generated during HRIM-measured swallows can be simultaneously analysed using pressure-flow analysis (PFA), an analytical method which unravels greater meaning from the complex sequence of pressure and impedance values than separate analyses of these measures [2–6]. The key to pressure-flow analysis is innovative software, which objectively derives the unique PFA measures of the neuromuscular mechanics of swallowing [7, 8]. Published studies in adults with pharyngeal dysphagia have shown that PFA measures and a global composite score of swallowing dysfunction, called the Swallow Risk Index (SRI), can discriminate normal from abnormal swallows [5, 6]. Furthermore, individual PFA variables can predict clinically and pathophysiologically relevant aspects of swallowing such as aspiration risk, the presence of postswallow residue, and circumstances of abnormal timing of pharyngeal bolus distension relative to propulsive contraction, a marker of poor oral containment and/or delayed swallow trigger [2–6].

The ability to perform multiple longitudinal, nonradiological HRIM measurements of swallowing function over time may have substantial clinical utility. Examples include neurodegenerative swallowing decline in motor neurone disease, stroke recovery, iatrogenic dysphagia in head, and neck radiotherapy or assessing interventions such as UES dilatation. The reliability of PFA software has been previously evaluated in two reports utilising HRIM studies performed in patients with dysphagia [7, 8]. These studies show that software can objectively derive PFA variables and the SRI with substantial to excellent inter- and intrarater agreement. Presently, the* test-retest* reliability of repeated HRIM recordings, involving reintubation with the same catheter system at a later time-point, is unknown. We therefore aimed to assess the intrarater and interrater agreement as well as test-retest reliability of PFA variables derived by multiple analysts from HRIM recordings obtained from the same individuals over two different studies.

## 2. Methods and Materials

### 2.1. Subjects

We report data based on pharyngeal HRIM studies performed in five healthy subjects across the age spectrum (2 males, aged 22–76 years; mean age 61 years). We performed the measurements in the Department of Anaesthesiology, University Hospital in Örebro, Sweden, and these were approved by the Central Ethics Review Board in Uppsala, Sweden (EC:Dnr 2013/251; approved on 26/06/2013). None of the participants reported any current or past symptoms of dysphagia or upper gastrointestinal diseases, smoked, or took any medications that could affect pharyngeal or esophageal function.

### 2.2. Protocol

We studied volunteers on two different occasions, approximately one week apart, and recorded pressure-impedance data using a 4.2 mm diameter catheter housing 36 circumferential pressure sensors (spaced 1 cm apart) and 18 impedance segments (Sierra Scientific Instruments, Inc., Los Angeles, CA). The catheter was calibrated in accordance with manufacturer's specifications. Catheter placement to straddle the entire pharyngoesophageal segment was performed without topical anaesthesia. Following a period of accommodation, participants swallowed 10 × 10 mL saline boluses on command that were administered via a syringe at >20 sec intervals. The Repeat Study was performed with the catheter positioned at the same level of insertion and boluses were administered identically.

### 2.3. Swallow Export and Randomisation

**Figure 1:**
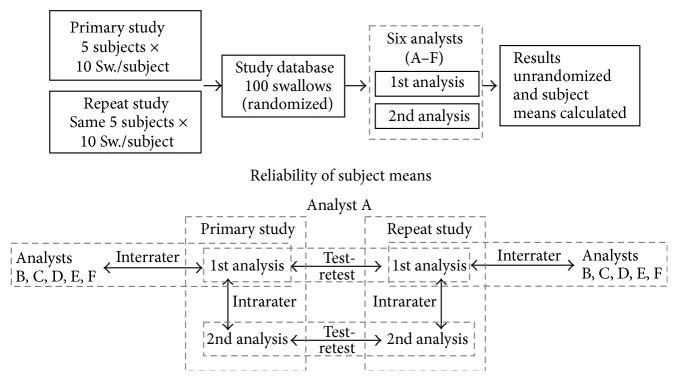
Study outline. The master swallow database comprised a total of 100 × 10 mL swallows (10 swallows × two measurements × 5 subjects) recorded in Manoview. Swallows were randomised and then six observers, who received identical training, performed repeat analyses of all swallows. Analyst data sets were then unrandomised and average values for each metric were calculated (i.e., Primary and Repeat Study means for each of the five subjects derived for each of Analyses 1 and 2).

The master swallow database for this reliability study comprised a total of 100 × 10 mL swallows (10 swallows × two measurements × 5 subjects) recorded in Manoview (Sierra Scientific Instruments, Inc., Los Angeles, CA). The acquisition system allowed export of raw pressure and impedance data for each swallow (5 sec window acquired at 100 samples per sec) in text format (.txt) for separate analysis. The deidentified swallows were randomised creating an analyst swallow database numbered from 1 to 100 (Figure 1).

### 2.4. Analysis Software

Swallows were consecutively analysed using a purpose designed software platform (*Aimplot2*; copyright Taher Omari, based in MATLAB version 8.5.0.197613, R2015a; The MathWorks Inc.). To operate the software, the analyst opened a colour pressure isocontour plot of each swallow file. With opening, the pressure and impedance data were automatically interpolated (Piecewise Cubic Hermite Interpolating Polynomial) to increase the data set to a 1 mm spatial resolution.

**Figure 2:**
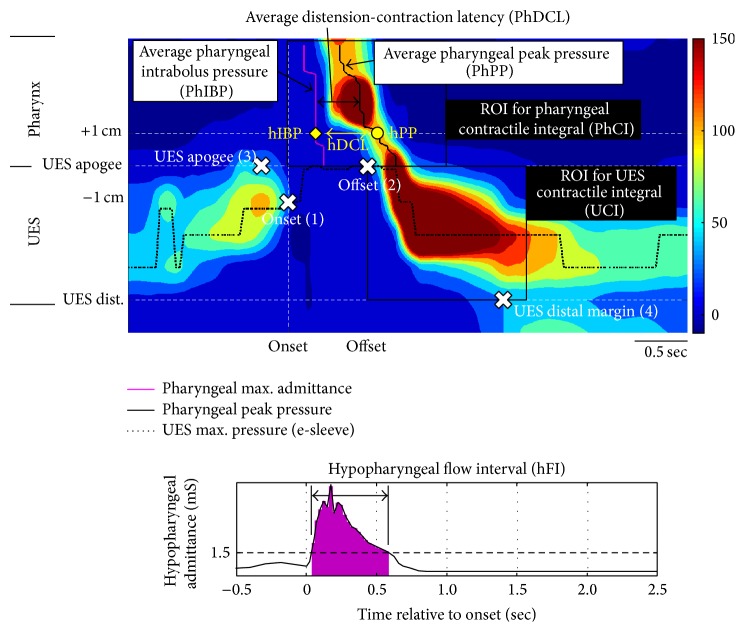
Software-derived variables of swallowing function. To operate analysis software, the analyst opened a colour pressure isocontour plot of each swallow file. Analysts then defined four space-time landmarks on the plot (white crosses). Guided by definition of these landmarks, the software then automatically generated values for swallow function variables measuring contractility (PhPP, hPP, and PhCI), intrabolus distension pressure guided by maximum admittance/nadir impedance (PhIBP, hIBP), flow timing (PhDCL, hDCL), and bolus presence (hFI).

The analysts then defined four space-time landmarks on the plot, as described in the following (see also Figure 2):

1. the time of onset of complete UES relaxation;
2. the time of offset of complete UES relaxation;
3. the apogee position of the UES high pressure zone, defined by visualisation of the orad movement of the UES high pressure zone to determine the highest position of the proximal edge of the high pressure zone during the swallowing event;
4. the distal margin position of the UES high pressure zone, defined by lowest position of the distal edge of the high pressure zone before and/or after swallow.

Guided by definition of these landmarks, the software then automatically generated values for a range of swallow function variables that the analyst copied to a spreadsheet template (Microsoft Excel; Microsoft Corporation, Redmond, WA).

### 2.5. Swallow Function Variables

Automatically derived swallow variables were separated into three subclasses: one, measures of contractility; two, measures of intrabolus distension pressure; and three, measures of flow timing. Finally, the Swallow Risk Index (SRI), a composite score of global dysfunction, was determined. We provide specific details of all variables in the following (see also Figure 2).

#### 2.5.1. Contractility

Contractility of the whole pharynx was determined for the pharyngeal stripping wave proximal of the UES apogee position using the average pharyngeal peak pressure (PhPP) and the pharyngeal contractile integral (PhCI) of pressures greater than 20 mmHg from onset of complete UES relaxation to 0.5 sec after offset of relaxation. A discrete hypopharyngeal peak pressure was also obtained at 1 cm proximal to the UES apogee position (hPP).

Using the* e-sleeve* method based on maximum axial UES pressures, basal UES pressure (UBP) was determined using the average pressure up to 0.25 sec prior to complete UES relaxation. Postrelaxation peak pressure (UPP) was determined by the maximum postrelaxation pressure up to 1 sec after relaxation offset. Finally, the UES contractile integral (UCI) was determined based on postrelaxation pressures greater than 20 mmHg up to 1 sec after relaxation offset.

#### 2.5.2. Intrabolus Distension Pressure

The intrabolus pressure variables measured in this study were for the most part derived using the principle of “pressure-flow analysis” in that impedance measurement (or its inverse called “admittance”) guided the position where intrabolus distension pressures should be measured [9–11]. During passage of a highly conductive bolus, the nadir impedance (or maximum* admittance*, expressed in millisiemens (mS), the unit of electric conductance) corresponds to the time and position where the lumen is most conductive. In normal circumstances this identifies the axial centre, or most distended part, of the intrabolus domain during transport [9–11]. Hence, pressure at nadir impedance (or maximum admittance) is an accurate measure of intrabolus distension pressure. Intrabolus distension pressure of the whole pharynx (PhIBP) was determined for the entire pharyngeal region proximal to the UES apogee position using the average pressure at nadir impedance. In addition, discrete hypopharyngeal intrabolus pressures (hIBP) were measured 1 cm proximal to the UES apogee position. Distension pressure within the UES region was determined based on the 0.25 s integrated UES relaxation pressure (UIRP). This is the median of all lowest pressures (contiguous or noncontiguous) recorded measured by the e-sleeve method over a 0.25 sec period [11, 12].

#### 2.5.3. Flow Timing

The temporal relationship between pharyngeal peak admittance and peak pressure defines the latency from maximum bolus distension to maximal contraction. The distension-contraction latency of the whole pharynx (PhDCL) was determined for the pharyngeal region proximal of the UES apogee position using the average time from peak admittance to peak pressure. In addition, a discrete hypopharyngeal distension-contraction latency (hDCL) was also obtained at 1 cm proximal to the UES apogee position. Hypopharyngeal Flow Interval (hFI) defining bolus dwell time in the hypopharynx during the swallow was determined at the level of the hypopharynx and based upon the total time that hypopharyngeal admittance exceeded the threshold of 15 mS, which, in a previous study, optimally defined postswallow luminal closure over the bolus tail [11].

#### 2.5.4. Swallow Risk Index

The SRI is derived by the formula: SRI = ((PhIBP*∗*hFI)/(PhPP*∗*[PhDCL + 1]))*∗*100. The SRI is a composite score capitalising on the observed directionality of four key swallow function variables when assessed in relation to increased dysfunction (as defined by aspiration on videofluoroscopy), namely, decreased contractile vigour (*lower* PhPP), higher intrabolus distension pressure (*higher* PhIBP), and aberrant timing (*longer* hFI and* shorter* PhDCL) [5, 6].

### 2.6. Analysts

Six observers repeat-analysed the study database (three medical officers, two speech and language pathologists, and one scientist; three with previous experience using PFA software). All observers received identical training in the use of the software and the identification of pressure landmarks to derive outcome measures. A demonstration video (30 min) and set of 10 practice swallows enabled the observers to develop a minimum level of understanding and competence in using the analysis software before proceeding to their formal analysis of the database swallows; then each observer performed repeat analyses of all swallows in their own time. Each observer returned two separate data sets of results from their 1st and 2nd analysis of the randomised database comprising 50 Primary Study swallows and 50 Repeat Study swallows. Analysed data sets were then unrandomised with average values for each metric calculated based on the 10 swallows recorded for each of the five subjects and each of the two studies (i.e., Primary and Repeat Study means for each of the five subjects derived for each of observer analysis runs 1 and 2).

### 2.7. Reliability Measures

We assessed the intrarater agreement (1st analysis versus 2nd analysis), interrater agreement (All Rater Combinations), and test-retest reliability (Primary Study versus Repeat Study) of subject means derived from the analysed data sets (Figure 1). Reliability measures were derived using the IBM SPSS statistics package Version 22 (IBM corporation, Somers, NY, USA). Absolute agreement of mean values was assessed using a two-way mixed model of intraclass correlation coefficient (ICC) based on single measures. For interrater comparisons, ICC was determined for Primary Study and Repeat Study using means from the 1st analysis run only. We interpreted the scale of intraclass correlations as follows: 0.00–0.20 =* slight agreement*; 0.21–040 =* fair*; 0.41–0.60 =* moderate*; 0.61–0.80 =* substantial*; and 0.81–1.00 =* excellent agreement*.

## 3. Results

All observers completed the repeat software-based analysis for all 100 swallows (i.e., total of 200 discrete analysis operations performed by each observer). The intrarater and interrater average intraclass correlation coefficients of swallow function variables showed* substantial* to* excellent* agreement across the board (Tables 1 and 2, resp.). Test-retest results were less reliable with agreement ranging from* slight* to* excellent* depending on the class of the swallow function variable examined. Contractility variables were less test-retest reliable than intrabolus pressure or bolus flow timing variables (Table 3).

Amongst contractility variables, preswallow UES basal pressure showed* excellent* agreement from Primary to Repeat Study and measures of UES postrelaxation contractile pressure showed* moderate* to* substantial* agreement, whilst agreement of measures of pharyngeal contractile pressure ranged from only* slight* to* substantial* (Table 3). Depending on the contractility variable, the average difference in the pharyngeal contractile pressure measurements from the Primary to Repeat Study ranged from 117 mmHg* higher* (UPP) to 143 mmHg* lower* (hPP) on Repeat (Table 4).

The test-retest agreement of measures of pharyngeal and UES intrabolus distension pressure was* substantial* (Table 3). Depending on the intrabolus pressure variable, the average difference in the distension pressure measurements from the Primary to Repeat Study ranged from 5 mmHg* higher* (hIBP) to 9 mmHg* lower* (PhIBP) on Repeat (Table 4). Test-retest reliability of all bolus flow timing measures was* substantial* to* excellent* (Table 3).

**Figure 3:**
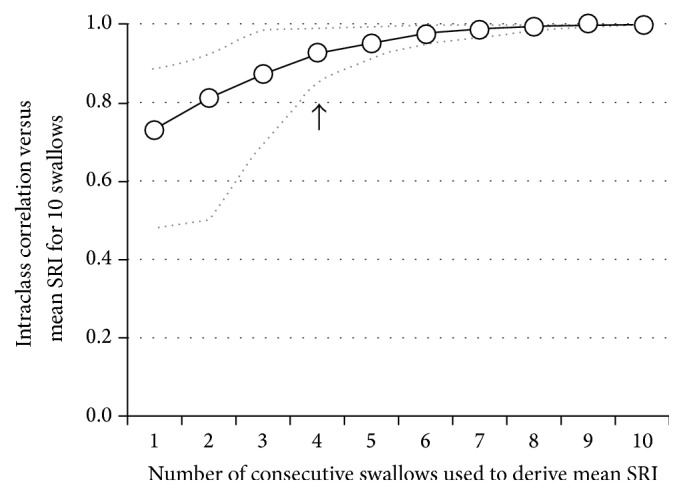
Intraclass correlations between the mean SRI using 1 to 10 consecutive swallows versus the all-swallow mean SRI. Data are based on 1st analysis results for Primary and Repeat Studies combined (10 measures, two per analyst). Analyst minimum and maximum ICC are grey dotted lines. Note:* excellent* reliability (ICC > 0.8) was found for all analyst results (i.e., when the grey dotted line representing minimum analyst ICC breaches the 0.8 threshold) when the SRI was based on the average of at least four consecutive swallows (arrow).

The Swallow Risk Index composite score, a global measure of swallowing dysfunction, showed* excellent* intra- and interrater agreement and* substantial* test-retest reliability (Tables 1–3) and the average difference in the SRI ranged from 2 units* higher* or 4 units* lower* on Repeat (Table 4). Using SRI calculation from 10 swallows as the benchmark, we performed a separate analysis to determine if the number consecutive swallows used influenced SRI reliability. Based on 1st analysis results for Primary and Repeat Studies combined (10 measures, two per analyst), the SRI derived from the first swallow demonstrated* moderate* to* excellent* agreement against the benchmark of 10-swallow average SRI (ICC range 0.48–0.88; mean 0.73). Agreement improved when increasing numbers of consecutive swallows were included (Figure 3). SRI based on the average of at least four consecutive swallows (ICC range 0.85–0.99, mean 0.92) achieved* excellent* agreement amongst all observers.

## 4. Discussion

Using data gathered in healthy individuals across the age spectrum, we evaluated the reliability of HRIM recordings and swallow function variables. Software-derived variables had high intra- and interrater agreement. However, the test-retest agreement of measurements taken in the same individuals 1 week apart was highly dependent upon the variable subtype measured, with intrabolus distention pressures and timing variables displaying the greatest degree of test-retest reliability.

The current study, conducted in healthy volunteers, confirms previous evaluations of swallows from patients and healthy subjects showing that software-based analysis of pharyngeal pressure and pressure-impedance recordings can be reliably analysed by different observers, even those with little or no experience with a HRM/HRIM procedure [7, 8, 13]. We propose that the high degree of reliability stems from the use of highly recognisable spatiotemporal landmarks and the use of objective, software-driven, analysis algorithms. These render to the background any methodological complexities, while at the same time deriving numerical measures easily comparable with reference ranges to detect abnormality and elucidate pathophysiology. It is important to note that the current study tested how reliably results could be generated, but not how reliably they are* interpreted*. Whilst analysts can make reliable measurements, the pathophysiological interpretation of these results is another matter entirely that we have not addressed in our study.

In practice, we have applied software-based PFA through derivation of average values for swallows captured following oral administration of a standardised volume and consistency bolus. In the current study, we compared average values derived from 10 × 10 mL liquid bolus swallows given to each subject during each study. In our view, liquid boluses represent the best system and observer test because they exhibit the greatest swallow to swallow variability due to low viscosity and the potential for air to influence the impedance recordings. Whilst we tested and confirmed high levels of interobserver and intraobserver agreement, our primary motivation for performing this study was to assess the test-retest reliability of the method.

The reliability of measurements made over repeated HRIM investigations is critically important to understand and quantify. This is because the ability to repeat investigations over time improves clinical utility through quantification of changes in swallowing function due to disease or following procedures/interventions. Our study shows that intraclass correlations of the test-retest comparisons were lower overall than the intra- and interrater comparisons. Hence, by repeating a study, we potentially introduce additional factors superimposed onto the analysis-related factors. This diminishes reliability even though every possible effort was made to standardise conditions that are system-related (e.g., acquisition settings; catheter processing; prestudy calibration; and postacquisition temperature compensation) and/or protocol-related (e.g., clinic room temperature; time of day; use of local anaesthetic or not; nares side; depth of catheter insertion; accommodation period; bolus volume, consistency, and temperature; and length of study).

Whilst inter- and intrarater agreement was almost uniformly excellent, we found that test-retest results were less reliable and the level of agreement differed substantially depending on the class of measurement. Measures that quantified the isometric pressures generated by the pharyngo-UES stripping contraction agreed least on a test-retest comparison. This was despite the fact that we used state-of-the-art circumferential pressure sensing. Indeed, between Primary and Repeat Study, the average absolute contractile pressure and differences exceeded 50 mmHg for most volunteers. Poor reliability of the pharyngeal measures of contractility in particular may relate to the fact that the actual location of the catheter within the right, left, or centre of the pharyngeal chamber and movement of the catheter shaft during the swallow was not controlled and could therefore have been different between studies. The pharyngeal constrictors and cricopharyngeus muscle contract asymmetrically and longitudinally as well as in the anteroposterior dimension [14, 15]. Hence, any longitudinal or axial movement of the catheter combined with subtle positioning differences could have changed the exertion of pressures onto the pressure sensing arrays, potentially altering both measured pressure and ability of observers to see landmarks clearly. Such factors are difficult to control. On the other hand, test-retest agreement of UES basal pressure and intrabolus distension pressures was* substantial* to* excellent*, suggesting that technical factors, such as thermal compensation, pressure drift, sensor failure, and calibration error, are unlikely to explain the selective impairment of the reliability being limited to active contractility measures during swallow.

Intrabolus distension pressures and measures such as distension-contraction latency, which define timing relationships between waveform peaks, were highly reliable in the test-retest analysis. We believe that this relates to the fact that distension pressures reflect pressures within “open distended chamber” during bolus flow and, therefore, are not subject to the influences of symmetry. These are furthermore less subject to errors in axial localisation because the bolus distension pressures are usually common over a greater axial distance. On the other hand, timing variables are least affected by these factors because latency measurements do not depend upon the pressure recording being accurate in absolute terms.

The fact that distension and timing measures are more “reliable” may explain why these measures often show significant differences in relation to pathology and bolus-type/consistency. In several populations, bolus distension pressures and flow timing variables appear to be the most critical variables required to differentiate pharyngeal dysphagia patients [2–6]. The Swallow Risk Index combines four key variables into a global composite score predictive of predisposition to aspiration risk. In the current study, the test-retest agreement of SRI was* substantial* and, additionally, we demonstrate that capture of at least four swallows is sufficient for reliable interpretation of the SRI. This number is consistent with the findings of a previous study of broad dysphagia patients which showed that at least four swallows are needed to derive an average SRI that falls consistently above or below our working diagnostic cut-off (SRI 15) [4].

Our study has some limitations, which are important to acknowledge. Whilst a large number of swallows were repeat-analysed (100 swallows, 200 data points for each metric per analyst), the database itself was derived from only five subjects who underwent repeat measurements (10 studies overall). By necessity, all test-retest comparisons were based on the test result averages. However, as all measurements were identically and simultaneously derived within the same swallows, we contend that the test-retest comparisons of ICC values amongst the different classes of variables (i.e., contractility versus flow timing versus distension pressure variables) are still very meaningful in their interpretation. We also note the very high intra- and interrater agreement in the current study; this finding is consistent with previous studies that tested intra- and interrater agreement of pressure-flow metrics [7, 8]. We did not assess different bolus volumes or consistencies because this would have resulted in an inordinate number of analysis permutations. We did not assess reliability in patients with dysphagia. This was due to the large number of liquid boluses that they would have been required to swallow and the critical need to undergo a repeat measurement, which was not feasible in dysphagia patients. Whilst our subjects were healthy, we purposefully included data from aged subjects to enable a wider distribution of results [3], albeit within the normal range.

In conclusion, HRIM based PFA measures of swallowing function can be derived using software-based analysis with* substantial* to* excellent* intra- and interrater agreement. System-related factors diminish the test-retest reliability of measures of swallowing function. Measures of flow timing, intrabolus distension pressure, and a global Swallow Risk Index showed* substantial* test-retest reliability, whilst measures of pharyngeal and UES contractility show* slight* to* excellent* test-retest reliability.

## Figures and Tables

**Table 1:** Intrarater reliability of 1st analysis versus 2nd analysis for the Primary and Repeat Studies. Data are average ICC [range ICC].

	ICC for 1st analysis versus 2nd analysis	
	Primary Study	Repeat Study
Contractility		
PhPP	0.98 [0.90–1.00]	0.93 [0.67–1.00]
PhCI	0.97 [0.91–1.00]	0.87 [0.51–0.98]
hPP	0.94 [0.84–0.99]	0.91 [0.71–0.98]
UBP	1.00 [1.00-1.00]	1.00 [0.99–1.00]
UPP	0.93 [0.75–1.00]	0.98 [0.89–1.00]
UCI	0.88 [0.71–0.96]	0.99 [0.97–1.00]
Intrabolus pressure		
PhIBP	1.00 [1.00–1.00]	0.97 [0.88–1.00]
hIBP	0.98 [0.95–1.00]	0.99 [0.97–0.99]
UIRP	0.99 [0.97–1.00]	1.00 [0.99–1.00]
Flow timing		
PhDCL	0.96 [0.79–1.00]	0.97 [0.91–1.00]
hDCL	0.85 [0.29–0.99]	0.93 [0.83–0.99]
hFI	0.99 [0.99–1.00]	0.99 [0.96–1.00]
Global function		
Swallow Risk Index	0.99 [0.94–1.00]	0.94 [0.80–1.00]

**Table 2:** Interrater reliability of all rater combinations for the 1st analysis of the Primary and Repeat Studies. Data are average ICC [range ICC].

	ICC for All Rater Combinations	
	Primary Study (1st analysis)	Repeat Study (1st analysis)
Contractility		
PhPP	0.98 [0.95–1.00]	0.95 [0.78–1.00]
PhCI	0.97 [0.92–1.00]	0.74 [0.31–1.00]
hPP	0.93 [0.77–1.00]	0.87 [0.73–0.98]
UBP	0.99 [0.97–1.00]	0.99 [0.96–1.00]
UPP	0.96 [0.86–1.00]	0.92 [0.70–1.00]
UCI	0.88 [0.66–0.98]	0.84 [0.38–1.00]
Intrabolus pressure		
PhIBP	1.00 [0.99–1.00]	0.97 [0.87–1.00]
hIBP	0.98 [0.89–1.00]	0.98 [0.87–1.00]
UIRP	0.99 [0.95–1.00]	0.97 [0.91–1.00]
Flow timing		
PhDCL	0.98 [0.93–1.00]	0.92 [0.75–1.00]
hDCL	0.93 [0.76–1.00]	0.77 [0.11–1.00]
hFI	0.99 [0.98–1.00]	0.98 [0.93–1.00]
Global function		
Swallow Risk Index	1.00 [0.99–1.00]	0.94 [0.75–1.00]

**Table 3:** Test-retest reliability of Primary Study versus Repeat Study for the 1st and 2nd analysis. Data are average ICC [range ICC].

	ICC Primary Study versus Repeat Study	
	1st analysis	2nd analysis
Contractility		
PhPP	0.22 [0.03–0.41]	0.24 [0.02–0.59]
PhCI	0.24 [0.05–0.42]	0.15 [0.05–0.24]
hPP	0.61 [0.41–0.93]	0.59 [0.23–0.96]
UBP	0.94 [0.93–0.95]	0.94 [0.93–0.96]
UPP	0.49 [0.43–0.63]	0.47 [0.28–0.57]
UCI	0.67 [0.61–0.72]	0.62 [0.54–0.67]
Intrabolus pressure		
PhIBP	0.73 [0.67–0.86]	0.75 [0.68–0.82]
hIBP	0.80 [0.63, 0.93]	0.80 [0.66–0.94]
UIRP	0.80 [0.71–0.88]	0.79 [0.65–0.88]
Flow timing		
PhDCL	0.86 [0.65–0.96]	0.89 [0.82–0.95]
hDCL	0.79 [0.70–0.84]	0.61 [0.15–0.84]
hFI	0.91 [0.89–0.93]	0.91 [0.84–0.95]
Global function		
Swallow Risk Index	0.75 [0.65–0.89]	0.80 [0.69–0.87]

**Table 4:** Test-retest change in measurements from Primary Study to Repeat Study in swallow function variables. Data are mean difference in the results of the six analysts with 95% confidence intervals of the difference shown in parentheses. Individual data for each study volunteer are shown (based on 1st analysis results only).

	Change from Primary Study to Repeat Study mean difference (5%, 95% confidence interval)				
	Volunteer 1	Volunteer 2	Volunteer 3	Volunteer 4	Volunteer 5
Contractility					
PhPP mmHg	84 (68, 99)	76 (65, 88)	−57 (−60, −63)	0 (−3, 2)	34 (24, 43)
PhCI mmHg·cm·s	−11 (−26, 5)	26 (16, 36)	−83 (−88, −77)	−63 (−69, −58)	−82 (−88, −77)
hPP mmHg	142 (96, 188)	143 (58, 227)	0 (−34, 35)	−3 (−6, 0)	88 (43, 132)
UBP mmHg	−21 (−22, −20)	1 (−3, 6)	0 (−1, 1)	−13 (−15, −10)	−8 (−14, −2)
UPP mmHg	−117 (−148, −85)	−88 (−99, −76)	−26 (−32, −20)	59 (−15, −11)	−43 (−50, −36)
UCI mmHg·cm·s	−89 (−108, −70)	−65 (−78, −52)	−55 (−67, −43)	83 (78, 87)	−4 (−26, 18)
Intrabolus pressure					
PhIBP mmHg	3 (2, 3)	−5 (−6, 3)	−2 (−3, −2)	−4 (−4, −3)	9 (6, 12)
hIBP mmHg	−3 (−4, −2)	−3 (−4, −2)	0 (−2, 3)	−5 (−6, −5)	8 (4, 11)
UIRP mmHg	−1 (−2, 0)	−3 (−3, −3)	−4 (−4, −3)	−4 (−5, −3)	6 (5, 7)
Flow timing					
PhDCL msec	−1 (−14, 11)	−13 (−24, 2)	54 (50, 58)	−5 (−7, −3)	−68 (−118, −20)
hDCL msec	−111 (−138, −83)	−59 (−99, −18)	27 (16, 38)	−10 (−22, 23)	−18 (−52, 17)
hFI msec	20 (2, 39)	5 (−19, 29)	−2 (−20, 16)	−19 (−34, −4)	130 (115, 144)
Global function					
Swallow Risk Index	0 (0, 0)	−2 (−3, −1)	0 (0, 0)	−1 (−1, −1)	4 (3, 6)
